# Distinct Transcriptional Responses of Skeletal Muscle to Short-Term Cold Exposure in Tibetan Pigs and Bama Pigs

**DOI:** 10.3390/ijms24087431

**Published:** 2023-04-18

**Authors:** Chunhuai Yang, Chunwei Cao, Jiali Liu, Ying Zhao, Jianfei Pan, Cong Tao, Yanfang Wang

**Affiliations:** 1 State Key Laboratory of Animal Nutrition, Institute of Animal Science, Chinese Academy of Agricultural Sciences, Beijing 100193, China; 2 Guangdong Laboratory for Lingnan Modern Agriculture, Guangzhou 510642, China; 3 Guangdong Provincial Key Laboratory of Malignant Tumor Epigenetics and Gene Regulation, Guangdong-Hong Kong Joint Laboratory for RNA Medicine, Sun Yat-Sen Memorial Hospital, Sun Yat-Sen University, Guangzhou 510275, China

**Keywords:** pig, cold exposure, transcriptome, skeletal muscle

## Abstract

Piglets are susceptible to cold, and piglet death caused by cold stress leads to economic losses in the pig industry in cold areas. Skeletal muscle plays a key role in adaptive thermogenesis in mammals, but the related mechanism in pigs is unclear. In this study, cold-tolerant Tibetan pigs and cold-sensitive Bama pigs were subjected to either a cold environment (4 °C) or a room temperature environment (25 °C) for 3 days. The biceps femoris (BF) and longissimus dorsi muscle (LDM) were collected for phenotypic analysis, and the BF was used for genome-wide transcriptional profiling. Our results showed that Tibetan pigs had a higher body temperature than Bama pigs upon cold stimulation. RNA-seq data indicated a stronger transcriptional response in the skeletal muscle of Tibetan pigs upon cold stimulation, as more differentially expressed genes (DEGs) were identified with the same criteria (*p* < 0.05 and fold change > 2). In addition, distinct pathway signaling patterns in skeletal muscle upon cold exposure were found between the breeds of pigs. Mitochondrial beta-oxidation-related genes and pathways were significantly upregulated in Tibetan pigs, indicating that Tibetan pigs may use fatty acids as the primary fuel source to protect against cold. However, the significant upregulation of inflammatory response- and glycolysis-related genes and pathways in the skeletal muscle of Bama pigs suggested that these pigs may use glucose as the primary fuel source in cold environments. Together, our study revealed the distinct transcriptional responses of skeletal muscle to cold stimulation in Tibetan pigs and Bama pigs and provided novel insights for future investigation of the cold adaptation mechanism in pigs.

## 1. Introduction

The external environmental temperature is a crucial factor in animal growth and the utilization of energy. According to a previous study, a cold environment is defined as a temperature below approximately 26 to 28 °C for weaned piglets and 18 to 20 °C for finishing pigs [1]. Cold stress always induces the incidence of diarrhea [2] and death in newborn piglets and reduces the performance of weaned pigs [3], causing serious economic loss to the pig industry. Although housing and practical husbandry could improve the impact of cold stress, efficient dietary energy utilization reduces metabolic heat production in pigs [4]. It has been well recognized that the lack of both functional uncoupling protein 1 (UCP1) and brown adipose tissue (BAT) in pigs [5,6,7] causes the cold sensitivity of modern domestic pigs. However, some pig breeds in China, such as the Tibetan pig inhabiting the Qinghai-Tibetan Plateau and the Min pig inhabiting northeastern China, are well recognized as having adapted to a cold environment. A better understanding of thermogenesis in response to cold stress in these cold-resistant breeds could be beneficial to pig welfare and reduce the economic effects of cold stress. Lin et al. investigated the potential mechanisms of cold adaptation in Tibetan pigs and found that acute cold stimulation (4 °C for 4 h) induces the expression of uncoupling protein 3 (UCP3) and the formation of beige adipocytes in adipose tissues of cold-adapted pigs, implying the key roles of adipose tissues in protecting against cold environments [8]. However, the mechanisms of thermoregulation in other organs need to be explored deeply.

Skeletal muscle, which accounts for approximately 40% of total body weight, makes a major contribution to the basal metabolic rate and is involved in body temperature regulation [9]. Shivering thermogenesis is the primary form of thermogenesis in skeletal muscle during acute cold exposure and is related to the activity of actin, myosin and ATP [10]. An increasing number of studies have indicated that in addition to shivering thermogenesis, non-shivering thermogenesis (NST), which involves sarcolipin (SLN)-mediated uncoupling of sarcoplasmic reticulum calcium ATPase (SERCA) [11,12] and UCP3-mediated uncoupling of mitochondria [13], plays a crucial role in adaptive thermogenesis in skeletal muscle. In rodents, SERCA-derived NST is an important compensation mechanism for BAT-dependent NST [14,15]. However, the role of SLN-mediated NST in the skeletal muscle of large mammals is still controversial [16], as the expression of *SLN* and *SERCA1A* did not increase in skeletal muscle from chronically cold-exposed (5 °C/−5 °C, 25 days) Altay sheep [10] and from both chronic (5 °C/−5 °C for 55 days) and acute (−15 °C/−24 °C for 3 days) cold-exposed Min pigs, a well-known cold-tolerant pig breed in China [17]. Due to its high expression in skeletal muscle and homology to UCP1, UCP3 was assumed to be linked to NST in skeletal muscle [13]. A recent study reported its uncoupled function in vivo [18]. UCP3 overexpression can result in increased oxidation of fatty acids in the skeletal muscle of mice [19,20,21,22]. Isolated soleus muscles from UCP3-overexpressing mice have a higher resting heat rate at a low temperature (20 °C), proving the thermogenic function of UCP3 [23]. However, suppression of UCP3 expression was observed in skeletal muscles from humans with prolonged exposure to a cold environment, suggesting the complex regulatory roles of UCP3 in thermogenesis [24]. Our previous study revealed the critical role of UCP3-mediated thermogenesis in cold adaptation in Tibetan pigs [8], but its thermogenic function in the skeletal muscle of pigs is unclear.

Here, we investigated the transcriptional response of skeletal muscles upon cold adaptation in cold-tolerant and cold-sensitive pig breeds. Weaned Tibetan pigs (TP) and Bama pigs were either placed in a cold chamber set at 4 °C, or kept at a room temperature (RT) of 25 °C for 3 days. The biceps femoris (BF) and longissimus dorsi muscles (LDM) from pigs of both breeds under both conditions were collected and subjected to histological analyses, including determination of muscle fiber size and myofiber type. The genome-wide gene expression levels in the BF of pigs of both breeds were measured by RNA-seq. Differentially expressed genes (DEGs) and pathways enriched with these genes upon cold treatment in both pig breeds were screened and analyzed.

## 2. Results

### 2.1. No Significant Changes in Myofiber Size and Type Were Observed in Skeletal Muscles from Cold-Exposed Tibetan Pigs and Bama Pigs

**Figure 1:**
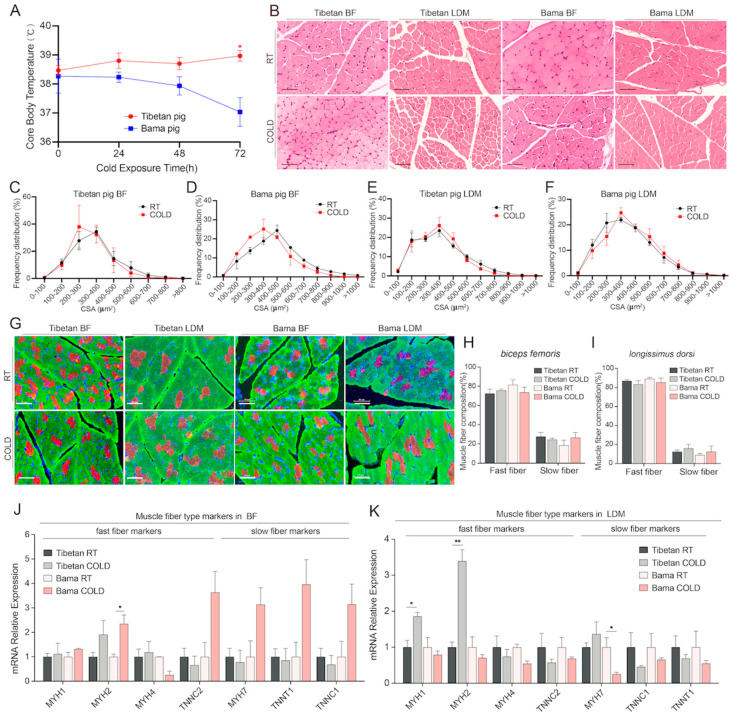
Effects of short-term cold exposure on the core body temperature, muscle fiber area distribution, and muscle fiber type composition in Tibetan pigs (TP) and Bama pigs (BP). (**A**) Core body temperature of TP and BP during short-term cold exposure. (**B**–**F**) H&E staining of tissue sections of the BF and LDM from TP and BP (**B**). Representative images are shown. Scale bar, 50 μm. The myofiber cross-sectional area distribution in the biceps femoris (BF) and longissimus dorsi muscles (LDM) of TP and BP was measured by Image J (n = 3 muscle samples) (**C**–**F**). (**G**–**I**) Immunofluorescence analysis of fiber type composition in the BF and LDM of TP and BP (**G**). The different myosin heavy chain isoforms are indicated by green (fast fibers) and red (slow fibers) fluorescence. Representative micrographs and the corresponding quantitative results (**H**,**I**) are shown (n = 3). Scale bar, 50 μm. (**J**,**K**) mRNA expression levels of fast and slow myofiber markers in the BF and LDM of TP and BP (n = 3). The results are presented as the mean ± standard error mean (SEM). * *p* < 0.05; ** *p* < 0.01.

To determine the effects of short-term cold exposure on skeletal muscle in Tibetan pigs and Bama pigs, three pigs of each breed were housed in a cold chamber (4 °C) for 3 days with ad libitum access to food and water, and another three pigs were kept at room temperature and served as controls. The core body temperature in the two groups of pigs was measured during cold exposure, and we found that the core body temperature of Tibetan pigs was significantly higher than that of Bama pigs after short-term exposure, indicating that Tibetan pigs had better cold adaptability (Figure 1A). It has been reported that changes in the fiber area [25] and fiber type occur in skeletal muscle after cold exposure [26]. To examine whether short-term cold exposure affects the histological features of skeletal muscle in the two pig breeds, we compared the slides of hematoxylin and eosin (H&E)-stained BF and LDM cross-sections from the RT and CD groups of pigs and assessed the changes in muscle fiber size (Figure 1B). Our data showed that the cross-sectional area (CSA) of muscle fibers in the two pig breeds was not significantly changed after a cold challenge (Figure 1C–F). To further determine whether short-term cold exposure affects the myofiber type, we quantified the muscle fiber type composition by immunostaining muscle cross-sections with antibodies specific for fast and slow fibers (Figure 1G). The composition of the myofiber type in the BF and LDM in the two breeds of pigs exhibited no appreciable difference after cold exposure (Figure 1H,I). Next, we measured the mRNA expression levels of several muscle fiber type marker genes (*MYH1*, *MYH2*, *MYH4*, *MYH7*, *TNNT1*, *TNNC1,* and *TNNC2*) in both types of skeletal muscle tissues from pigs of both breeds by qPCR. As shown in Figure 1J, although an increase in the expression of slow fiber marker genes was observed in the BF of cold-exposed Bama pigs, the difference was not statistically significant. In addition, the expression of the fast fiber marker *MYH2* was significantly induced only in the BF of Bama pigs (Figure 1J). In contrast, the expression of the fast fiber markers *MYH1* and *MYH2* was significantly upregulated only in the LDM of Tibetan pigs (Figure 1K).

### 2.2. A Stronger Transcriptional Response of Skeletal Muscle upon Cold Stimulation Was Observed in Tibetan Pigs

**Figure 2:**
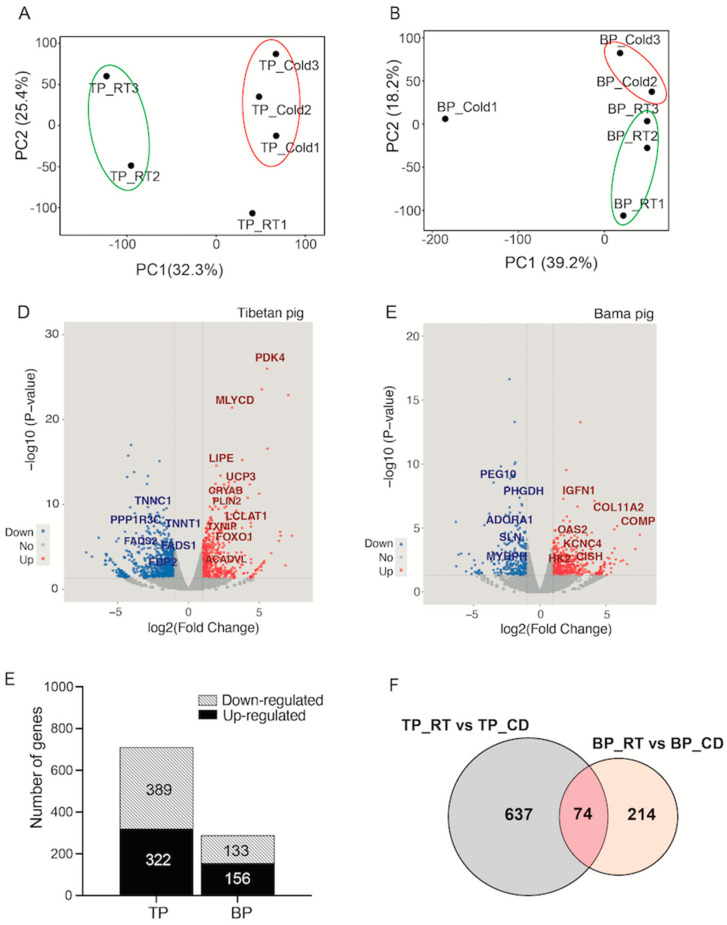
RNA-seq analysis of differential expressed genes (DEGs) in the BF of TP and BP under room temperature (RT) or cold (CD) conditions. (**A**,**B**) PCA of samples from TP and BP. (**C**,**D**) Volcano plots showing significance on the y-axis (−log 10 *p*-value) plotted against the gene expression ratio (log_2_ FC) on the x-axis; the *p* < 0.05 significance level is indicated by gray dashed horizontal lines. (**E**) The numbers of upregulated (black) and downregulated (striped) DEGs between Tibetan and Bama pigs. (**F**) Venn diagram based on the number of DEGs (FC > 2, *p*-value < 0.05).

To explore the molecular profile alterations in skeletal muscle occurring in response to cold exposure, global RNA-seq analysis of the BF from pigs of both breeds before/after cold exposure was performed. The results of principal component analysis (PCA) showed that the samples from the cold-exposed group were separated from those from the RT group in both pig breeds (Figure 2A,B), implying changes in the transcriptome in cold-exposed BFs. To obtain a broad overview of the transcriptional changes in skeletal muscle in both breeds of pigs, volcano plots were generated based on the *p*-value and fold change (Figure 2C,D). A total of 711 DEGs were identified in Tibetan pigs by the criteria of fold change (FC) > 2 and *p*-value < 0.05, of which 322 were significantly upregulated and 389 were significantly downregulated (Supplementary Table S2). However, only 288 DEGs were found to exhibit significant changes, as defined by the same criteria, in Bama pigs, with 155 upregulated and 133 downregulated (Supplementary Table S2 and Figure 2E). These data indicate that Tibetan pigs seem to be more sensitive to short-term cold exposure and show greater transcriptional plasticity. As shown in Figure 2F, only 74 overlapping genes were identified between the pig breeds, 637 DEGs were uniquely identified in Tibetan pigs, and 214 DEGs were identified only in Bama pigs.

### 2.3. Common Molecular Features of Cold-Exposed Skeletal Muscles from Both Pig Breeds

**Figure 3:**
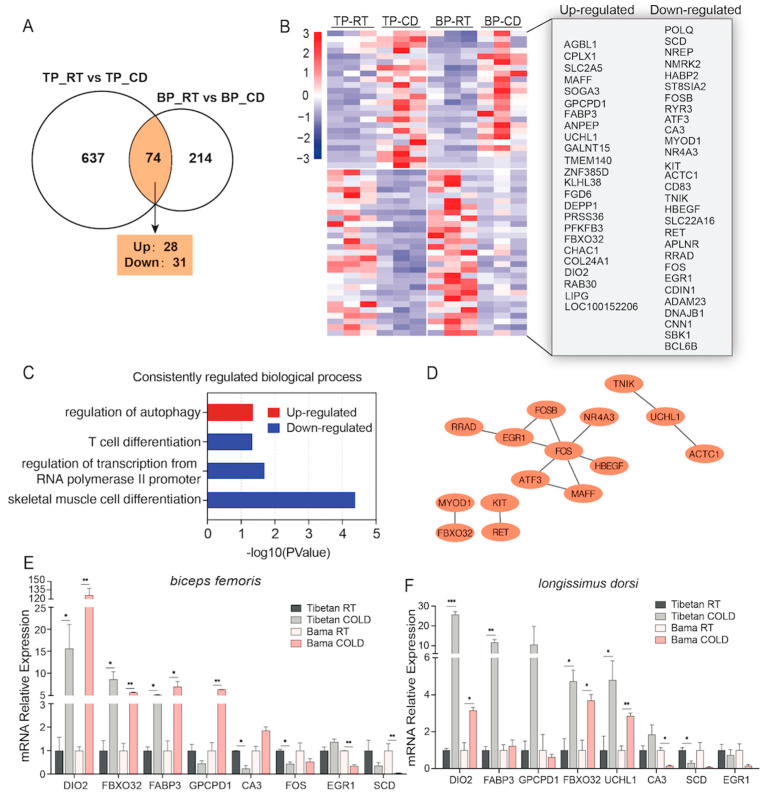
Functional enrichment analysis and verification of the genes commonly regulated by cold exposure in TP and BP. (**A**)Venn diagram showing the number of commonly regulated DEGs between the two breeds. (**B**) Heatmap and detailed list of genes with the same expression trends. (**C**) All gene ontology (GO) terms enriched with the commonly regulated genes. (**D**) The protein-protein interaction (PPI) network of the commonly regulated genes. (**E**,**F**) qPCR validation of commonly regulated genes in the BF (**E**) and LDM (**F**); these genes are involved in glucose and lipid metabolism, as well as skeletal muscle cell differentiation. All values are presented as the mean ± SEM. * *p* < 0.05; ** *p* < 0.01; *** *p* < 0.001.

Breed-independent molecular features of the response of skeletal muscle to cold stimulation were further identified by exploring the 74 overlapping DEGs. Fifty-nine of these DEGs showed the same expression trend in both breeds of pigs after cold exposure; 28 were significantly upregulated, and 31 were significantly downregulated (Supplementary Table S1 and Figure 3A). The heatmap clearly demonstrated the consistent expression profiles of these genes in both pig breeds (Figure 3B). Gene ontology (GO) analysis was performed, and we found that the upregulated DEGs were markedly enriched only in the GO term regulation of autophagy. Moreover, the downregulated DEGs were found to be enriched in terms of skeletal muscle cell differentiation, regulation of transcription from the RNA polymerase Ⅱ promoter and T cell differentiation, etc. (Figure 3C). We also constructed a protein-protein interaction (PPI) network with these commonly regulated DEGs. GO annotations of skeletal muscle development were extracted from the PPI network (Figure 3D). Furthermore, the RNA-seq results were further confirmed by qPCR for several genes, including *DIO2*, the key gene of cold-induced thermogenesis; *FABP3*, *GPCPD1,* and *SCD*, which participate in lipid metabolism; and *FBXO32*, *FOS*, *EGR1,* and *UCHL1*, which mediate skeletal muscle differentiation (Figure 3E). Significantly induced expression of *DIO2* and *FBXO32* was also observed in the LDM of pigs of both breeds (Figure 3F).

### 2.4. Significant Induction of Immune Response-Related Genes Was Observed Only in Cold-Exposed Skeletal Muscles from Bama Pigs

**Figure 4:**
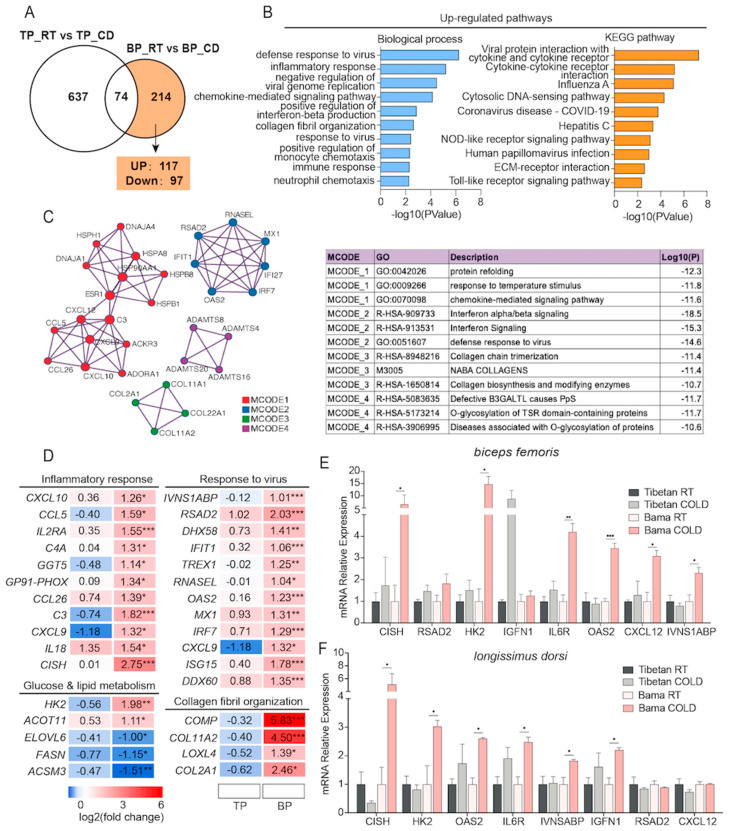
Short-term cold exposure uniquely regulates inflammatory response-related genes in BP. (**A**) Venn diagram showing the number of uniquely regulated DEGs in BP. (**B**) GO terms and KEGG pathway analyses of upregulated DEGs. (**C**) A PPI network of uniquely regulated genes in BP. The molecular complex detection (MCODE) algorithm was applied to identify densely connected network components. Each MCODE network was assigned a unique color. The three highest scoring terms with the highest by *p*-value were retained as the functional descriptions of the corresponding components, as shown in the table under the network diagram. (**D**) Heatmaps showing the relative changes in the expression of genes involved in the inflammatory response, the formation of collagen fibril organization, and glucose and lipid metabolism (n = 3). (**E**,**F**) qPCR validation of genes involved in the inflammatory response in the BF (**E**) and LDM (**F**). All values are presented as the mean ± SEM. * *p* < 0.05; ** *p* < 0.01; *** *p* < 0.001.

We then investigated the 214 DEGs that were uniquely identified in Bama pigs (Supplementary Table S1 and Figure 4A) to identify the Bama pig-specific responses to cold exposure. GO analysis suggested that the upregulated genes were enriched in the biological processes defense response to virus, inflammatory response, negative regulation of viral genome replication, chemokine-mediated signaling pathway, positive regulation of interferon-beta production, etc. (Figure 4B), while the downregulated genes were enriched in L-serine biosynthetic process, extracellular matrix organization, protein refolding, labyrinthine layer blood vessel development, response to heat, etc. (Figure S1). The induction of immune response pathways was further confirmed by Kyoto Encyclopedia of Genes and Genomes (KEGG) pathway analysis (Figure 4B). In addition, the following pathways were inhibited: biosynthesis of amino acids; glycine, serine, and threonine metabolism; cysteine and methionine metabolism; the MAPK signaling pathway; and protein processing in the endoplasmic reticulum (Figure S1). Furthermore, the interactions among DEGs were analyzed with the molecular complex detection (MCODE) algorithm. The largest cluster was associated with the response to temperature stimuli and chemokine-mediated signaling pathways. Genes in the MCODE_2 network were involved in interferon signaling and the defense response to viruses. Genes in the MCODE_3 and MCODE_4 networks participated in collagen biosynthesis and O-glycosylation of proteins (Figure 4C). Notably, all these MCODE networks were related to the immune response. The heatmap of representative genes in each MCODE cluster is shown in Figure 4D. qPCR analysis was also used to confirm the induction of specific genes, including *OAS2*, *CXCL12*, *IVNS1ABP*, *IL6R,* and *IGFN1*, in BF and LDM samples from Bama pigs (Figure 4E,F). Notably, HK2, a major rate-limiting enzyme of the glycolytic pathway, was significantly upregulated in both the BF and LDM of cold-exposed Bama pigs, indicating that Bama pigs may maintain their core temperature by utilizing extracellular glucose during short-term cold exposure.

### 2.5. Fatty Acid β-Oxidation-Related Genes Were Significantly Induced in Cold-Exposed Tibetan Pigs

**Figure 5:**
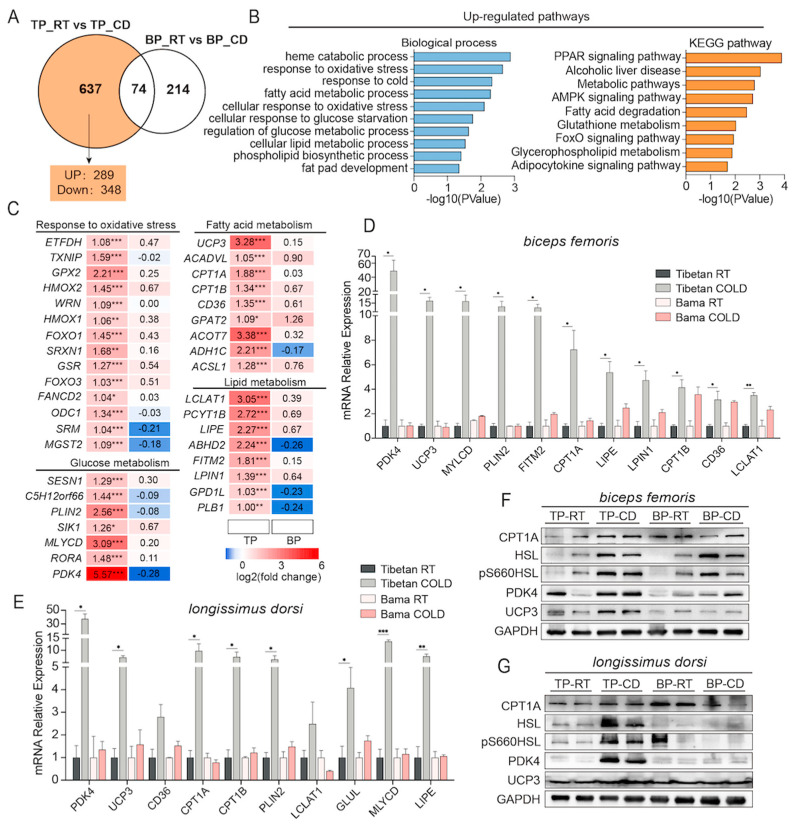
Short-term cold exposure uniquely regulates lipid catabolism-related genes in TP. (**A**) Venn diagram showing the number of uniquely regulated DEGs in TP. (**B**) GO and KEGG pathway analyses of upregulated DEGs. (**C**) Heatmaps showing the relative changes in the expression of genes involved in the response to oxidative stress and in glucose and lipid metabolism (n = 3). (**D**,**E**) qPCR validation of randomly selected genes that were uniquely upregulated in the BF (**D**) and LDM (**E**) of TP. (**F**,**G**) Expression of the fatty acid uptake- and lipolysis-related proteins CPT1A, HSL (LIPE), pS660HSL, PDK4, and UCP3 in the BF (**F**) and LDM (**G**) in TP and BP. All values are presented as the mean ± SEM. * *p* < 0.05; ** *p* < 0.01; *** *p* < 0.001.

We further investigated the 637 DEGs that were specifically found in the skeletal muscle of cold-exposed Tibetan pigs, of which 289 were upregulated and 348 were downregulated (Supplementary Table S1 and Figure 5A). The upregulated genes were enriched in the GO terms heme catabolic process, response to oxidative stress, response to cold, fatty acid metabolic process, cellular response to glucose starvation, etc. (Figure 5B). KEGG pathway analysis further confirmed this observation (Figure 5B). In addition, the downregulated DEGs were found to be enriched In the GO terms ventricular cardiac muscle tissue morphogenesis, canonical Wnt signaling pathway, transition between fast and slow fiber, etc., and the KEGG pathways hypertrophic cardiomyopathy, dilated cardiomyopathy, circadian rhythm, PI3K-Akt signaling pathway, protein digestion and absorption, etc. (Figure S2). We also created a heatmap with these genes to clearly demonstrate the specific response of skeletal muscle to cold in Tibetan pigs (Figure 5C). qPCR analysis and western blotting were used to further validate the RNA-seq results. In detail, *CD36*, *CPT1A*, *CPT1B,* and *UCP3*, which are responsible for the transport of fatty acids to the cytoplasm and mitochondria, were significantly upregulated in the BF (Figure 5D) and LDM (Figure 5E), indicating that the uptake of fatty acids in the skeletal muscle of Tibetan pigs might be enhanced. The increased expression of *PDK4* and *HSL* in both types of skeletal muscle in Tibetan pigs was further confirmed at both the mRNA level (Figure 5D,E) and protein level (Figure 5F,G), indicating that the substrates of cellular oxidative metabolism were converted from glucose to fatty acids and that the lipolysis and mitochondrial uptake of fatty acids were enhanced. In addition, the expression of *LCLAT1* and *LPIN1*, which are involved in the glycerophospholipid metabolism pathway, was also significantly upregulated. Our results demonstrated that Tibetan pigs may protect themselves from cold environments via the decomposition of fatty acids in skeletal muscles. The marked upregulation of UCP3 revealed its participation in skeletal muscle thermogenesis in Tibetan pigs.

## 3. Discussion

Here, we investigated the effect of cold stimulation on the skeletal muscles of Tibetan pigs and Bama pigs. We found that the muscle fiber size was not markedly altered by short-term cold exposure in either pig breed, consistent with observations in rats [27] and newborn pigs [28]. However, after prolonged cold stimulation (8–10 weeks), muscle fiber atrophy was observed in hibernating mammals such as hamsters [27]. It has been reported that long-term cold exposure (over a month) has an effect on the transition of fiber type and increases the proportion of slow fibers in piglets [26,29]. However, neither a significantly increased number of slow fibers nor induced expression of the related genes were observed in either type of skeletal muscle in either pig breed. In contrast, a slight but significant induction of the fast fiber marker genes *MYH1* and *MYH2* was observed in the LDM of Tibetan pigs. The discrepancy might be due to the shorter cold treatment time in this study, and we believe that prolonged cold exposure could significantly change the phenotype of myofibers.

Organisms can adapt to environmental changes by reprogramming gene expression [30]. Previous studies have reported that cold-tolerant animals, such as deer mice [31] and cold-tolerant chickens [32], exhibit enhanced transcriptional plasticity to cope with cold environments. Consistent with these observations, we found that skeletal muscle from Tibetan pigs exhibited a stronger transcriptional response in the cold environment than that from Bama pigs, as we observed in adipose tissues [8].

Our transcriptome data revealed that short-term cold exposure increased the expression of fatty acid transport- and mitochondrial beta-oxidation-related genes in the skeletal muscle of Tibetan pigs, while fatty acid biosynthesis-related genes were found to be downregulated, indicating a large change in fuel selection during short-term cold stress. Moreover, the significant induction of PDK4, a key regulator of glucose and fatty acid metabolism, in the skeletal muscle of Tibetan pigs further confirmed that Tibetan pigs utilize fatty acids as the primary fuel to support energy metabolism. With the enhancement of the lipid oxidation capacity, additional physiological changes are required to ensure efficient tissue O_2_ delivery. Our results showed enhancement of heme catabolic processes (increased gene expression of *HMOX1* and *HMOX2*), indicating that blood O_2_ affinity might be elevated in Tibetan pigs during short-term cold exposure. In addition, we also noted upregulation of UCP3, which was proven to participate in non-shivering thermogenesis in the adipose tissue of cold-tolerant pigs [8], implying the existence of UCP3-mediated thermogenesis in the skeletal muscle of Tibetan pigs but not in that of Bama pigs. In contrast, there were no significant changes in the expression of the above-mentioned genes in the skeletal muscle of Bama pigs, but GO terms related to the immune response were enriched, consistent with our observation in cold-exposed adipose tissues [8]. Furthermore, according to a previous study on genomic differences between Tibetan pigs and Chinese domestic pigs, the dominant genes in Chinese domestic pigs were related to the immune response (for example, the biological processes of immune response, inflammatory response, positive regulation of leukocytes, etc.) [33], which might explain the higher sensitivity of immune genes to respond to cold stress.

The key role of glycerophospholipids in adipose tissue thermogenesis has recently been reported in rodents [34,35,36,37]. Interestingly, we found that glycerophospholipid metabolism-related genes, such as *PCYT1B*, *GPAT2*, *LCLAT1*, *LPIN1*, *GPCPD1*, etc., were upregulated in the BF of cold-exposed Tibetan pigs, suggesting the activity of glycerophospholipid metabolism in Tibetan pigs. Our previous study showed changes in glycerophospholipid levels in adipose tissues from *UCP1* knock-in pigs, whose thermoregulation ability was significantly improved [38,39]. It is of interest to investigate the content and role of glycerophospholipids in the skeletal muscle of pigs of both breeds after cold exposure in future studies.

In fact, large mammals rely more heavily on shivering thermogenesis upon cold stimulation [40]. Here, we only focused on the transcriptional response of skeletal muscles to cold exposure in both Tibetan and Bama pigs, and the contribution of shivering to thermogenesis in both pig breeds was not precisely measured. Many of the lipid metabolism-related genes that were upregulated in the skeletal muscles of Tibetan pigs after cold stimulation fuel shivering, suggesting that stronger shivering occurred in Tibetan pigs; however, the exact difference in shivering between the pig breeds needs to be evaluated by electromyography (EMG) or energy expenditure assays.

**Figure 6:**
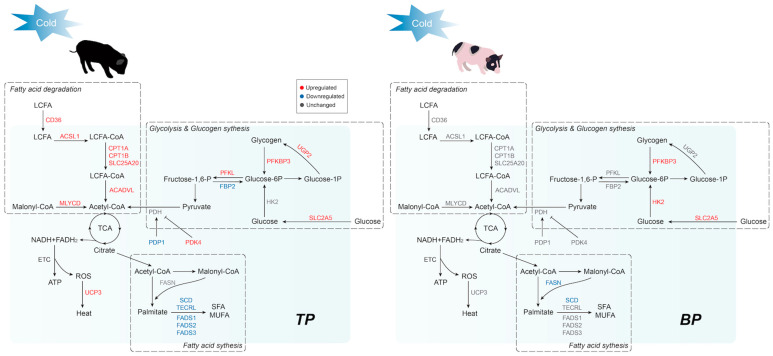
Schematic diagram of the distinct transcriptomic responses to short-term cold exposure in the BF of Tibetan and Bama pigs; pathways and genes involved in fatty acid, glucose, and glycogen metabolism are shown in the diagram.

In conclusion, our study revealed the distinct adaptive responses of skeletal muscle in Tibetan and Bama pigs to short-term cold exposure. Tibetan pigs exhibited a stronger transcriptional response and might exhibit greater utilization of fatty acids in skeletal muscle to protect against cold stress (Figure 6). Although more molecular mechanisms need to be further investigated, our study provided new data for a better understanding of the cold adaptation mechanisms in the skeletal muscle of pigs and paved the way for the discovery of potential targets to reduce cold stress-mediated mortality in piglets.

## 4. Materials and Methods

### 4.1. Animals and Experimental Design

The pigs used in this study had ad libitum access to a commercial pig diet (nutrient levels according to the NRC) and water throughout the experimental period. All experiments involving animals were performed according to the procedures approved by the Institutional Animal Care and Use Committee of the Institute of Animal Science, Chinese Academy of Agriculture Sciences (IAS2020-21). Bama pigs were provided by the Beijing Farm Animal Research Center affiliated with the Institute of Zoology, Chinese Academy of Science, and Tibetan pigs were provided by Hebei Linong Farm Co., Ltd. (Chengde, China).

Six Tibetan (male, 3.23 ± 0.20 kg) and six Bama (male, 3.45 ± 0.11 kg) weaned pigs, five weeks of age, were used in this study. Groups of three Tibetan pigs and three Bama pigs were established and housed in a cold chamber (3 d, 4 °C). The other three Tibetan pigs and Bama pigs were group-housed at room temperature (3 d, 25 ± 3 °C) and served as controls. Body temperature was measured by a rectal probe connected to a digital thermometer (Yellow Spring Instruments, Yellow Spring, OH, USA) every 24 h. After the cold challenge experiment, pigs were euthanized with an intravenous injection of sodium pentobarbital (Sigma, St. Louis, MO, USA) (drug dosing based on body weight) and slaughtered, and biceps femoris and longissimus dorsi muscle samples were collected immediately.

### 4.2. RNA Preparation and Quantitative Real-Time PCR

Total RNA was extracted from skeletal muscle tissues using RNAiso (Takara, Tokyo, Japan) in accordance with the manufacturer’s instructions. The quality and purity of total RNA were assessed using a spectrophotometer (NanoDrop 2000, Thermo Fisher Scientific, Waltham, MA, USA) at 260 and 280 nm. The absorption ratios (260/280 nm) of all samples were between 1.80 and 2.00. cDNA was synthesized by the Primer Script RT Reagent Kit with gDNA Eraser (Takara, Tokyo, Japan). Messenger RNA levels were measured by qPCR using a Quant Studio 3 system (Thermo Fisher Scientific, Waltham, MA, USA). Gene-specific primers were designed online by Primer 3 (version 4.1.0) (https://primer3.ut.ee/ (accessed on 14 December 2021)) and are listed in Supplementary Table S2. The PCR protocol was as follows: 95 °C for 5 min, followed by 40 cycles of 95 °C for 5 s and 60 °C for 34 s. Relative gene expression levels were calculated using the 2^−ΔΔCt^ (threshold cycle difference) method. GAPDH was used as the housekeeping gene.

### 4.3. RNA-Seq and Screening of Differentially Expressed Genes

Sequencing library preparation and RNA-seq were conducted at Shanghai Personal Biotechnology Co., Ltd. RNA samples with high purity (OD260/280 ≥ 2.0) and high integrity (RIN > 8) were used for cDNA library construction. More detailed information on cDNA library construction, sequencing of PE libraries, quality control, read mapping, and CPM calculations can be found in a previous study [8]. DEGs were identified as those meeting the criteria of *p* < 0.05 and FC > 2.0.

### 4.4. Bioinformatic Analysis

PCA was performed by the “gmodels” function in R Studio. Volcano plots were generated by the “ggplot2” function in R Studio. GO and KEGG analyses were completed using the “DAVID 2021” Functional Annotation Tool (https://david.ncifcrf.gov/home.jsp (accessed on 25 April 2022)). Pathways with *p* < 0.05 were regarded as statistically significant. PPI and MCODE networks of the DEGs were analyzed using the online Metascape database (https://metascape.org/gp/index.html#/main/step1 (accessed on 27 April 2022)). A heatmap was generated at https://www.bioinformatics.com.cn (accessed on 30 April 2022).

### 4.5. Immunoblotting

Skeletal muscle tissues were lysed in T-PER Tissue Protein Extraction Reagent (Thermo Fisher Scientific, Waltham, MA, USA) supplemented with a protease and phosphatase inhibitor cocktail (Roche, Indianapolis, IN, USA). Protein samples were boiled at 100 °C for 10 min, and proteins were then separated by electrophoresis on an SDS-PAGE gel (EpiZyme, Beijing, China). The separated proteins were transferred onto nitrocellulose membranes (Merck Millipore, Billerica, MA, USA), which were blocked with 5% skim milk for 2 h at room temperature and incubated with antibodies against PDK4 (1:2000, Proteintech, Wuhan, China), CPT1A (1:2000, Proteintech, Wuhan, China), UCP3 (1:2000, ABclonal, Wuhan, China), HSL, p-HSL (S660) (1:2000, CST, Danvers, MA, USA), Total OXPHOS (1:5000, Abcam, Cambridge, UK). An anti-GAPDH antibody (1:7500, CST, Danvers, MA, USA) was used to analyze the expression of GAPDH as the loading control. The membranes were incubated with HRP-conjugated secondary antibodies and visualized by enhanced chemiluminescence on a FluorChem M Fluorescence Imaging System (Tanon 5200, Tanon Science & Technology Co., Ltd., Shanghai, China).

### 4.6. Histology

Samples of skeletal muscle tissues were obtained and fixed with 4% paraformaldehyde, dehydrated using a graded ethanol series, and embedded in paraffin. Five micrometer-thick sections were finally cut on a microtome and mounted on slides. Multiple tissue sections were prepared and stained with H&E to analyze the CSA of each muscle fiber. For each sample, three nonconsecutive sections were processed for any given stain. Images were acquired using a microscope (DS-RI2; Nikon, Tokyo, Japan). At least five randomly selected nonoverlapping images were acquired for each cross-section, and the CSA of almost all muscle fibers (except for fibers with blurred outlines that could not be recognized) in each image was measured.

### 4.7. Immunofluorescence Staining

Immunofluorescence staining was performed using anti-fast myosin skeletal heavy chain (1:200, Abcam, Cambridge, UK) and anti-slow myosin skeletal heavy chain (1:200, Abcam, Cambridge, UK) antibodies. All sections were mounted using Fluoroshield media with DAPI (Abcam, Cambridge, UK). Images were acquired using a microscope, and quantification of the muscle fiber type composition was performed by Image J.

### 4.8. Statistical Analysis

GraphPad Prism (Version 9.0.0, La Jolla, CA, USA) was used for statistical analysis. Statistical comparisons between two groups were made using the unpaired, two-tailed Student’s t test. In all comparisons, the levels of statistical significance were set at * *p* < 0.05; ** *p* < 0.01 and *** *p* < 0.001. All data are presented as the mean ± standard error mean (SEM).

## Data Availability

The data presented in this study are available on request from the corresponding author. The data are not publicly available due to the fact that we are conducting further experiments.

## Supplementary Materials

The following supporting information can be downloaded at: https://www.mdpi.com/article/10.3390/ijms24087431/s1.
