# Supplementary material for: Distinct Transcriptional Responses of Skeletal Muscle to Short-Term Cold Exposure in Tibetan Pigs and Bama Pigs
Source: Int J Mol Sci. 2023 Apr 18;24(8):7431. doi: 10.3390/ijms24087431 (PMC10139196; doi:10.3390/ijms24087431)
Supplement: Supplementary file 1 [file ijms-24-07431-s001.zip › Supplementary Figures.pdf]

Figure S1. Gene Ontology and KEGG pathway analysis of BP uniquely down-regulated DEGs.

Figure S2. Gene Ontology and KEGG pathway analysis of TP uniquely down-regulated DEGs.

Figure S1

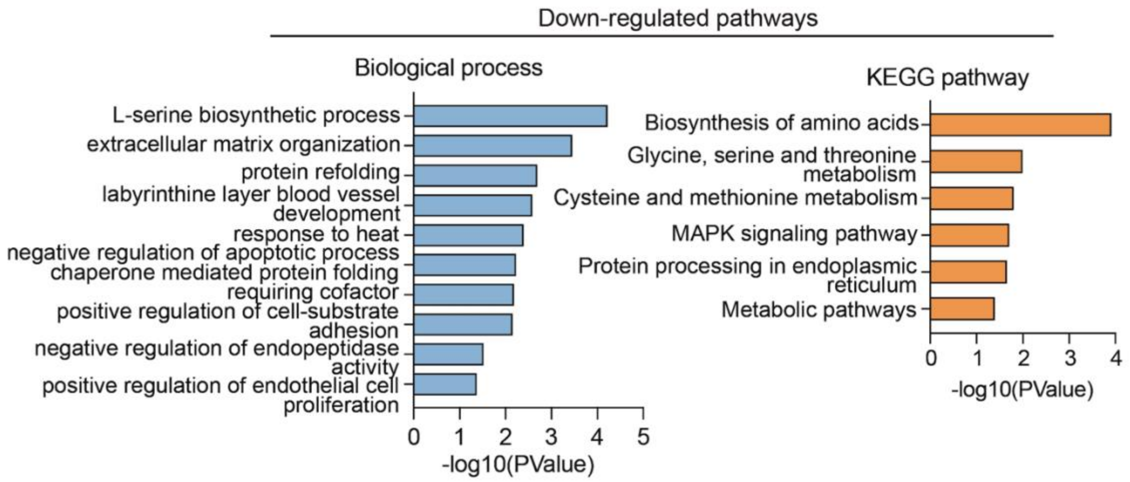

Down-regulated pathways
Biological process
L-serine biosynthetic process
extracellular matrix organization
protein refolding
labyrinthine layer blood vessel development
response to heat
negative regulation of apoptotic process
chaperone mediated protein folding requiring cofactor
positive regulation of cell-substrate adhesion
negative regulation of endopeptidase activity
positive regulation of endothelial cell proliferation
0 1 2 3 4 5
-log10(PValue)
KEGG pathway
Biosynthesis of amino acids
Glycine, serine and threonine metabolism
Cysteine and methionine metabolism
MAPK signaling pathway
Protein processing in endoplasmic reticulum
Metabolic pathways
0 1 2 3 4
-log10(PValue)

Figure S2

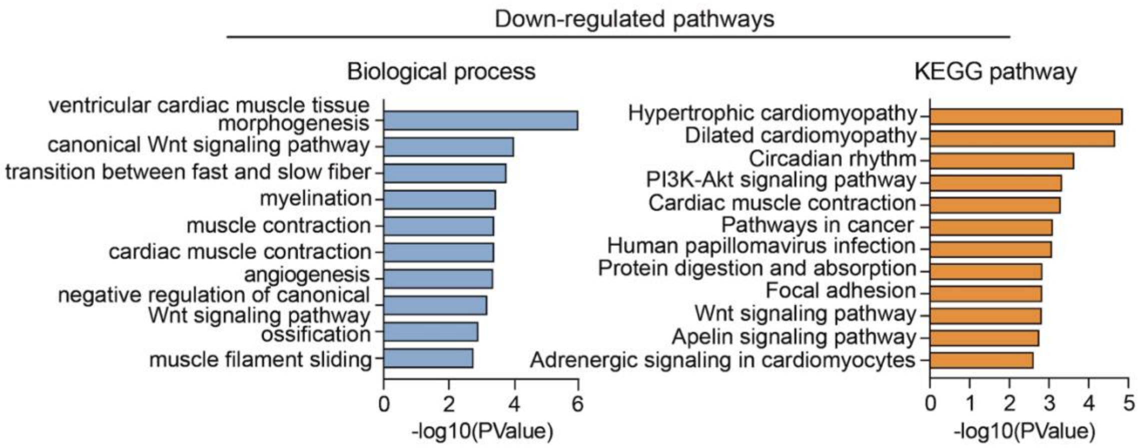
Down-regulated pathways
Biological process
ventricular cardiac muscle tissue morphogenesis
canonical Wnt signaling pathway
transition between fast and slow fiber
myelination
muscle contraction
cardiac muscle contraction
angiogenesis
negative regulation of canonical Wnt signaling pathway
ossification
muscle filament sliding
0
2
4
6
-log10(PValue)
KEGG pathway
Hypertrophic cardiomyopathy
Dilated cardiomyopathy
Circadian rhythm
PI3K-Akt signaling pathway
Cardiac muscle contraction
Pathways in cancer
Human papillomavirus infection
Protein digestion and absorption
Focal adhesion
Wnt signaling pathway
Apelin signaling pathway
Adrenergic signaling in cardiomyocytes
0
1
2
3
4
5
-log10(PValue)
